# Beyond Reporting: A Qualitative Approach to Managing Ethical Incidents in Healthcare

**DOI:** 10.1155/jonm/8870121

**Published:** 2026-03-08

**Authors:** Paula Järvisalo, Monika von Bonsdorff, Kaisa Haatainen, Marja Härkänen

**Affiliations:** ^1^ Department of Nursing Science, University of Eastern Finland, Yliopistonranta 8, Kuopio, 70210, Finland, uef.fi; ^2^ School of Business and Economics, University of Jyväskylä, Seminaarinkatu 15, Jyväskylä, 40014, Finland, jyu.fi; ^3^ Research Centre for Nursing Science and Social and Health Management, Kuopio University Hospital, Wellbeing Services County of North Savo, Kuopio, Finland, uef.fi

**Keywords:** artificial intelligence, ethical incident, healthcare, incident reporting, management, nurse

## Abstract

**Background:**

Patient safety incident reporting systems foster a nonblaming culture and support organizational learning. By incorporating an ethical dimension, these systems can support nursing managers in addressing ethical incidents, strengthen healthcare professionals’ ethical competence and conduct, and promote the delivery of high‐quality patient care.

**Aim:**

This study aimed to describe the types of reported ethical incidents, outline the actions and measures implemented to prevent recurrence, and reflect on how our findings inform the organization of ethical incident prevention management.

**Design:**

This retrospective register study was conducted using an artificial intelligence‐based closed data analysis program and inductive content analysis. The standards for reporting qualitative research (SRQR) checklist was used.

**Research Context:**

Data were collected using the HaiPro system at a university hospital in Finland from 2018 to 2021. During this time, 3615 patient safety incident reports were submitted, of which 579 (16%) were categorized as ethical incidents and subsequently analyzed.

**Ethical Considerations:**

Ethical requirements were met. According to the Finnish ethical guidelines, an ethical review was unnecessary for this register study. The research permit was provided by the participating organization.

**Results:**

We found two main categories: (1) ethical incidents, with subcategories of professional conduct and patient care management and (2) actions and preventive measures, emphasizing continuous improvement, enhancing professional competence, fostering professional communication and conduct, improving data management, and enhancing patient care through the reporting system.

**Conclusions:**

Cultivating an ethical culture through the strategic management of ethical incidents is vital. An efficient structure for handling such incidents can improve decision‐making, ensure accountability, support professionals, and reduce their burden, ultimately enhancing care quality.

**Implications:**

This study provides valuable insights for nursing managers on effectively addressing ethical incidents. Their role is pivotal in shaping an ethical culture through strategic leadership, promoting educational initiatives, and supporting the implementation of ethical practices.

## 1. Background

Ethical principles, such as autonomy, beneficence, nonmaleficence, and justice, guide healthcare practice [1] and are universally recognized across healthcare settings [2]. These principles shape the ethical competence and conduct of the nursing profession through laws, regulations, and standards, including the International Council of Nurses’ (ICN) Code of Ethics for Nurses [3] and the World Health Organization’s (WHO) ethical guidelines [4]. The ICN Code of Ethics for Nurses promotes high ethical standards globally and offers a framework for decision‐making and practice. It outlines nurses’ ethical responsibilities to patients, the profession, and society, emphasizing their integrity, professionalism, and compassionate care [3]. Similarly, WHO ethical principles guide healthcare practices and policies to ensure equity, transparency, and respect for human rights, fostering decisions that benefit all populations, especially the vulnerable [4].

Ethical incidents arise in healthcare because of various factors [5]. An ethical incident can be called an ethical dilemma, challenge, or conflict. In a dilemma or challenge, a professional must choose between two equally unsatisfactory alternatives for patient care [6]. Ethical conflicts can arise from inadequate patient care or insufficient professional collaboration and may be internal, related to personal values, or interpersonal, involving disagreements with colleagues [7]. Ethics consultation is considered essential for addressing issues related to patient communication, consent, autonomy, conflicts between economic and medical interests, and professional and organizational issues [8]. This study defines ethical incidents as deviations from ethical competence and conduct.

Ethical conduct is shaped by working conditions, workloads, administrative systems, and staffing levels [9]. Maintaining ethical conduct and thereby ensuring high‐quality care depends on ethical competence [9, 10], which is nurtured through education and organizational support [7]. Ethical competence equips nurses to navigate the ethical incidents they encounter effectively [11], enhancing their resilience [12], alleviating the ethical burden, and preventing moral distress [13].

Ethical competence and conduct thrive in an organizational culture that fosters open interprofessional communication, collaboration, and reflection on ethical incidents, free from the fear of harmful consequences [5, 14]. Such a culture encourages learning from incidents, enabling healthcare professionals to address ethical challenges clearly and confidently [15]. In an ethically supportive culture, professionals feel empowered, which enhances job satisfaction and retention rates [16] while promoting high‐quality patient care [17]. Strengthening this culture through discussions, ethics education, and supportive policies is essential [12].

If ethical incidents are not adequately addressed, they can lead to suffering for those involved [7, 18], causing moral distress in nurses [5, 18] and threatening their professionalism and dignity [7]. This, in turn, deteriorates the quality of care [18]. Strengthening ethical competence among healthcare professionals and students is therefore crucial [5], especially as challenges persist in ensuring that nursing students fully grasp and apply ethical principles in practice [19].

### 1.1. Patient Safety Incident Reporting: Promoting an Ethical Organizational Culture and Management

A patient safety incident reporting system, such as the Critical Incident Reporting System (CIRS), supports organizational learning by documenting incidents to identify areas for improvement, prevent recurrence, and highlight successes [20]. For optimal effectiveness, the system must be user‐friendly, provide actionable analytical insights, and facilitate comprehensive discussions among multiprofessional teams [21]. Integrating an ethical dimension into the system enables identifying, addressing, and resolving ethical incidents while fostering broader ethical discussions within healthcare organizations [8]. These systems allow healthcare professionals to report ethical incidents anonymously without fear of sanctions. This approach helps identify deficiencies in ethical behavior and underscores unethical conduct as a risk to patient care quality. In doing so, it can strengthen organizational ethics, enhance patient safety, and improve employee satisfaction [8, 22]. In Finland, ethical competence and conduct are supported through the web‐based patient safety incident reporting system HaiPro [22], which has been widely used since 2007 to manage patient safety incidents. The system is built on the principles of accessibility, anonymity, voluntariness, confidentiality, promoting openness, a nonblaming approach, and organizational learning. These principles foster a positive safety culture through transparent communication and continuous improvement. Ethical incident reporting was introduced in response to feedback on mistreatment. In 2017, a specific event type addressing ethical competence and conduct was added. This includes the following subcategories: (1) violation of patient autonomy; (2) violation of patient privacy; (3) discrimination; (4) causing unnecessary suffering; (5) inappropriate behavior; (6) restriction on treatment; (7) other; and (8) not selected. Reporters and managers document incidents, suggest preventive measures, and propose follow‐up actions in free‐text fields [23].

The role of a nursing manager is crucial in managing patient safety incident reports, coordinating subsequent interventions [24], and shaping outcomes for professionals and the workplace through proactive efforts to enhance staff satisfaction [25]. Nursing managers can enhance nursing competencies by fostering a positive organizational climate, providing opportunities for professional development, and ensuring nurses’ workplace well‐being. This, in turn, improves care quality, strengthens workforce commitment and retention, and fosters more effective interprofessional relationships [24]. Additionally, by promoting open conversations, nursing managers support ethical conduct, which helps reduce the burden on nurses [12]. Organizing and developing care protocols and practices, such as ethical incident prevention in healthcare, requires systematic management efforts. In the context of organizing ethical incident prevention, this could entail healthcare professionals collaboratively shaping and fostering a participatory approach to ethical management. A recent study concluded that HaiPro is an effective tool for managing ethical issues and promoting ethical practices at the unit level [22]. However, the perspective of organizing the management of ethical incident prevention at the organizational level remains understudied.

This study examined ethical incidents reported in the HaiPro system to identify their nature and outline actions and measures implemented to prevent recurrence from both professional and managerial perspectives. It also explored strategies for managing ethical incidents that support decision‐making among healthcare professionals and promote ethical patient care. Our interpretation of the results aims to provide insights into organizing the management of ethical incident prevention in healthcare. The analysis was conducted using an artificial intelligence (AI) closed data analysis program and inductive content analysis, making this study one of the first to apply AI‐assisted qualitative methods in this context. The findings aim to enhance understanding of managing ethical incidents in a nonblaming manner, emphasizing how healthcare organizations can support professionals in upholding ethical values, responsibilities, and accountability in practice. These insights provide valuable guidance for management strategies and educational planning.

## 2. Aim

This study aimed to describe the types of reported ethical incidents, outline the actions and measures implemented to prevent a recurrence, and reflect on how our findings inform the organization of ethical incident prevention management.

The research questions were as follows:

1. What types of ethical incidents are reported in healthcare?
2. What actions and preventive measures have been proposed or implemented to prevent the recurrence of ethical incidents in healthcare?
3. How does the patient safety incident reporting system with an ethical dimension enhance patient care?

## 3. Materials and Methods

### 3.1. Design

This retrospective register study was conducted with an AI closed data analysis program and inductive content analysis. The standards for reporting qualitative research (SRQR) checklist was used to ensure rigor and transparency.

### 3.2. Research Context

Data were collected using the HaiPro system at a university hospital in Finland from 2018 to 2021. During this time, 3615 patient safety incident reports were submitted, of which 579 (16%) were categorized as ethical incidents.

### 3.3. Data Analysis

These reports consist of structured and free‐text data. AI was used to analyze free text, offering efficiency, speed, and the reduction of human bias for objective interpretation. It can also identify subtle patterns and anomalies that may be overlooked in manual analyses, thereby providing deeper insights into ethical incidents [26]. The program used was Aiwo, an effective text analytics tool for categorizing text‐based data [27]. It integrates AI with qualitative research processes using natural language processing (NLP) techniques, such as Bidirectional Encoder Representations for Transformers (BERT), which rely on deep learning and pretrained language models fine‐tuned for tasks such as text categorization [28]. Aiwo is data‐driven, without predefined keywords, ensuring objective analysis and autonomous categorization of data into distinct themes [29].

The original data were securely transmitted from the participating organization to Aiwo Ltd in Excel. Aiwo personnel uploaded the reports to the system. Specific incidents were identified for analysis using an ethical incident filter. After AI categorization, the provided entries were transferred to Microsoft Excel and structured as in the original reports, including event descriptions, recurrence prevention suggestions, proposed actions, and implementation descriptions. They were then reviewed by the first author, with meaningful impressions simplified, coded, and documented in Excel using inductive content analysis [30]. All AI‐generated categories were critically reviewed by the first author with reflexive consideration, including reflection on potential biases, assumptions, and the authors’ influence on interpretation. During the data analysis, the research group held regular discussions to collaboratively make decisions and address challenges. This approach ensured that the analysis remained grounded in the data and that the process was transparent and methodologically rigorous. The simplified data were transferred to Microsoft Word, grouped into AI‐based categories, and structured as they appeared in the original incident reports. The data were then categorized by comparing the differences and similarities and organized into seven subcategories and two main categories. Table 1 presents an example of this analytical process.

**TABLE 1:** An example of the analysis process.

Event description				
AI‐determined main category: treatment				
AI‐determined subcategory: medicines				
Inductive content analysis				
Original impression	Simplified impression	Code	Subcategory	Main category
“A nurse in the recovery room, who had treated me unprofessionally throughout my stay there, set up my IV to flow freely with a strong pain medication that is usually administered in a carefully controlled and monitored manner through a dispenser. The medication flowed freely on my way from the recovery room to the traumatology ward. Eventually, a nurse noticed that the medication was flowing freely instead of through the dispenser and informed me of the error. Gradually, I began to feel better, but I remained deeply shaken, crying hysterically and panicking. I had already endured an emotionally distressing recovery room experience, and now this medication error made matters worse. I was left wondering if the nurse who had mistreated me in the recovery room had intentionally set up the medication incorrectly or if it had been a mistake due to carelessness.”	The patient reported unprofessional conduct and a possible intentional medication error by a recovery room nurse, resulting in an overdose of pain medication.	Carelessness in medication	Professional conduct	Reported ethical incidents
“The patient arrived by ambulance from another hospital to the ward for treatment without a pain pump and was EXTREMELY in pain upon arrival. Five nurses transferred the patient from the stretcher to the bed using a transfer board, but the patient screamed in pain. Their arms were too painful to touch at all. The patient was also cold and sweaty. Pain medication was sent with the ambulance from the sending hospital ward, but it hadn’t been given to the patient, despite the severe pain. This means the patient endured the entire transfer without any pain relief.”	The patient was transferred by ambulance from another hospital, and despite experiencing pain during the transfer and the fact that pain medication had reportedly been provided to the ambulance, the patient had not received any medication and was in severe pain upon arrival.	Inadequate pain medication	Patient care management	

### 3.4. Ethical Considerations

Ethical approval was not required under Finnish ethical regulations [31] or the research organization’s Ethics Committee (2022), as the study utilized only registered data. The participating organization approved the research permit in September 2022. The original data contained indirectly identifiable information, and anonymization was required prior to processing. Anonymization was conducted by the program administrator. The results were presented to prevent the identification of the originating units, and all data handling followed responsible research practices. Owing to the nature of the incident report data, they cannot be made publicly accessible. An AI‐based language model (ChatGPT) was used to assist with language editing. The authors take full responsibility for the content. Good scientific practice was adhered to throughout this study.

## 4. Results

AI categorized the data into four main categories, with each entry potentially linked to multiple categories, resulting in 1190 connections at the main category level and 2378 at the subcategory level. Of the 579 original reports, 440 (76%) were classified under the main category of “Working,” 405 (70%) under “Practices,” 215 (37%) under “Treatment,” and 130 (22%) under “Setting.” Detailed category information is presented in Table 2.

**TABLE 2:** AI‐based categories with the corresponding number of free text associations.

Working *n* = 440		Practices *n* = 405		Treatment *n* = 215		Setting *n* = 130	
Nurses	*n* = 238	Information flow and communication	*n* = 206	Medicine	*n* = 69	Rooms	*n* = 73
Physicians	*n* = 225	Instructions and guidance	*n* = 130	Operations and procedures	*n* = 57	Recovery room	*n* = 22
Emergency department	*n* = 103	Data management and protection	*n* = 86	Intravenous infusion	*n* = 39	Inpatient ward	*n* = 14
Schedules	*n* = 93	Meetings	*n* = 56	Medication	*n* = 33	Machines	*n* = 12
Workload	*n* = 80	Prescriptions and recommendations	*n* = 56	Surgery	*n* = 27	Outpatient clinic	*n* = 12
Infants and children	*n* = 52	Documentation	*n* = 34	Mortality and palliative care	*n* = 26	Pharmacies	*n* = 7
Responsibilities	*n* = 37	Practices and protocols	*n* = 25	Anesthesia	*n* = 26	Hygiene	*n* = 7
On‐call staff	*n* = 36	Trainings	*n* = 22	Follow‐up care	*n* = 25	Patient record systems	*n* = 5
Work shifts	*n* = 36	Patient records	*n* = 22	Intensive care	*n* = 25	Identification wristbands	*n* = 5
Reports	*n* = 32	Development	*n* = 17	Tests and diagnoses	*n* = 14	Bed capacity	*n* = 3
Calling	*n* = 29	Discharge	*n* = 16	Nutrition	*n* = 13	Storage and cabinets	*n* = 1
Surgeons	*n* = 25	Inspection	*n* = 12	Substances	*n* = 11		
Diligence	*n* = 21	Monitoring	*n* = 12	CCU and ICCU	*n* = 10		
Staffing ratio	*n* = 19	Referrals	*n* = 11	Dosages	*n* = 8		
Errors	*n* = 17	Work induction	*n* = 7	Medication lists	*n* = 8		
COVID‐19	*n* = 13	Forms	*n* = 6	Blood transfusions	*n* = 1		
Older adults	*n* = 12	Isolations	*n* = 5				
Perceiving matters	*n* = 11	Medical prescriptions	*n* = 4				
Secretaries	*n* = 8	Letters	*n* = 2				
Homecare	*n* = 6						
IT support	*n* = 1						
Dictations	*n* = 1						
Oversights	*n* = 1						
Total	*n* = 1096		*n* = 729		*n* = 392		*n* = 161

Within the “Working” category, 23 subcategories were identified, comprising 1096 separate free texts. The most common subcategories were “Nurses,” “Physicians,” “Emergency Department,” “Schedules,” and “Workload.” For “Practices,” 19 subcategories were identified, encompassing 729 separate free texts, with the most frequent subcategories being “Information Flow and Communication,” “Instructions and Guidance,” “Data Management and Protection,” “Meetings,” and “Prescriptions and Recommendations.” In the “Treatment” category, 16 subcategories were identified, totaling 392 separate free texts. The most common subcategories were “Medicine” and “Operations and Procedures.” For “Setting,” 11 subcategories were identified, covering 161 separate free texts, with “Rooms” being the most frequently mentioned. AI also identified connections between categories. The most frequently mentioned subcategories, each representing 10% or more of all original free texts, along with their associated categories and frequencies, are summarized in Table 3.

**TABLE 3:** The most frequently mentioned subcategories, along with associated categories.

Working
Nurses 41%
Physicians (*n* = 129)
Information flow and communication (*n* = 125)
Instructions and guidance (*n* = 63)
Physicians 39%
Nurses (*n* = 129)
Information flow and communication (*n* = 81)
Emergency department (*n* = 65)
Emergency department 18%
Physicians (*n* = 65)
Nurses (*n* = 55)
Medicines (*n* = 30)
Schedules 16%
Workload (*n* = 65)
Nurses (*n* = 47)
Physicians (*n* = 44)
Workload 14%
Schedules (*n* = 65)
Nurses (*n* = 48)
Physicians (*n* = 36)
Treatment
Medicine 12%
Nurses (*n* = 49)
Physicians (*n* = 45)
Emergency department (*n* = 30)
Operations and procedures 10%
Nurses (*n* = 31)
Physicians (*n* = 25)
Schedules (*n* = 15)
Practices
Information flow and communication 36%
Physicians (*n* = 81)
Nurses (*n* = 79)
Instructions and guidance 22%
Nurses (*n* = 65)
Physicians (*n* = 61)
Information flow and communication (*n* = 50)
Data management and protection 15%
Physicians (*n* = 46)
Nurses (*n* = 43)
Information flow and communication (*n* = 39)
Meetings 10%
Information flow and communication (*n* = 39)
Nurses (*n* = 17)
Physicians (*n* = 14)
Prescriptions and recommendations 10%
Physicians (*n* = 65)
Nurses (*n* = 48)
Instructions and guidance (*n* = 23)
Setting
Rooms 13%
Nurses (*n* = 50)
Physicians (*n* = 36)
Information flow and communication (*n* = 27)

**FIGURE 1:**
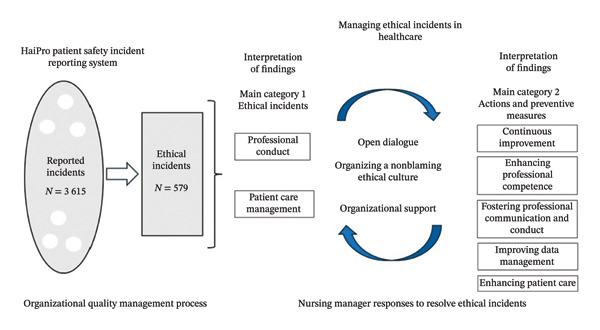
Managing ethical incidents in healthcare.

To deepen the insights provided by AI, further analysis focused on the first 100 most relevant free‐text entries from each subcategory. This analysis identified two main categories (1) ethical incidents, with subcategories of professional conduct and patient care management and (2) actions and preventive measures, with subcategories of continuous improvement, enhancing professional competence, fostering professional communication and conduct, improving data management, and enhancing patient care. These categories outlined the results. The order of presentation corresponds to the research questions (RQ1, RQ2, and RQ3). Selected quotations from the original reports illustrate the findings, with their corresponding main categories and subcategories indicated in parentheses. Figure 1 illustrates the management of ethical incidents in healthcare based on the results of this study.

### 4.1. Reported Ethical Incidents

#### 4.1.1. Professional Conduct

The reports highlighted ethical incidents involving various healthcare personnel, including nurses, doctors, housekeepers, and secretaries. Key issues included limited resources, such as inadequate staffing and expertise and insufficient orientation, leading to employees performing beyond their qualifications and resulting in errors in decision‐making, diagnostics, and medication management. Unclear responsibilities added to professionals’ confusion. Challenges in professional conduct were noted, including poor communication and unprofessional behavior toward colleagues, patients, and families, with some incidents involving persistent misconduct. A lack of collegial support was also reported.

“The ward was staffed with only four nurses. Two nurses were called to assist another ward due to an emergency. Simultaneously, multiple acute situations requiring nursing attention arose. Even after the two nurses returned, staffing levels remained insufficient for the demands.” (Working, Nurses)

#### 4.1.2. Patient Care Management

Ethical incidents involving vulnerable patient groups, including children, adolescents, individuals with mental health conditions, critically ill patients, end‐of‐life patients, and older adults, were frequently reported. Misplacement in unsuitable units caused delays, suboptimal care, and a lack of specialized expertise. Patients also faced inadequate respect and age‐appropriate treatment. End‐of‐life care concerns included unclear Do Not Attempt Resuscitation (DNAR) directives, inappropriate treatments, unnecessary transfers, and inadequate pain management. The challenges in data management and information flow included unclear directives, protocol deviations, documentation errors, privacy breaches, and compromised care. Improper room assignments raised also privacy concerns. These issues undermined care quality, burdened professionals, and contributed to patient suffering and stress among healthcare personnel.

“The patient was started on a morphine infusion due to an acutely deteriorating condition, with a dose that was very high considering the patient’s age and overall condition.” (Working, Older Adults)

### 4.2. Actions and Preventive Measures for Ethical Incidents

#### 4.2.1. Continuous Improvement

Continuous improvements in patient care quality involved investigating ethical incidents and fostering a learning culture through discussions. These investigations aimed to prevent future occurrences by identifying contributing factors, such as reviewing patient records and engaging with the involved personnel, patients, and families. These findings highlight the need for targeted improvements in several areas. Discussions on ethical incidents and preventive strategies were held with the employees involved and extended to multidisciplinary team meetings. Ethical incidents were also addressed in Moral Case Deliberation (MCD) meetings, offering a structured platform for reflecting on complex cases.

“A discussion with the nurse who treated the patient, as well as a team meeting within the department, was held to ensure that such treatment of patients does not occur in the future.” (Practices, Meetings)

#### 4.2.2. Enhancing Professional Competence

To promote ethical, high‐quality patient care, addressing the identified needs should focus on workforce management, ensuring adequate competence, and supporting the well‐being of professionals. Workforce management must ensure sufficient staffing, effective resource allocation, strategic scheduling, and balanced workload to enhance efficiency, reduce errors, and satisfy healthcare demands. Ensuring competence involves continuous training and regular assessments, particularly in care evaluations, patient interactions, and support for vulnerable groups. Up‐to‐date guidelines and collaborative practices between experienced and novice professionals are essential for sharing and developing skills. Supporting professionals’ well‐being is key to reducing stress, preventing ethical incidents, and maintaining high‐quality care while safeguarding mental health.

“The issue was discussed with the colleague in question, and procedural training was offered. It is important to ensure that temporary staff have adequate skills. Orientation procedures are being revised.” (Practices, Work Induction)

#### 4.2.3. Fostering Professional Communication and Conduct

These reports emphasized that strengthening professional collaboration and communication and maintaining professionalism are essential for ethical conduct. Ethical responsibility extends beyond patient care and requires active engagement within the work community and commitment to interdisciplinary teamwork. Effective task management and willingness to assume additional responsibilities are key to fostering collaboration and meeting a team’s dynamic needs. Clear and efficient communication ensures smooth information flow and optimal patient care, particularly in critical situations, with standardized protocols and attention to family communication. Professional behavior is expected in all interactions, with discretion and mutual respect maintained for patients and colleagues. In misconduct cases, thorough investigations and apologies were conducted, reinforcing a commitment to a respectful professional environment.

“Nurses have the right to receive support from the on‐call doctor regarding patient‐related issues and treatment matters. Cooperation should be smooth and collegial, as this is a workplace. A nurse should not fear or hesitate to call the on‐call doctor.” (Working, Physicians)

#### 4.2.4. Improving Data Management

Effective decision‐making in healthcare requires active patient consultation and prioritizing the patient’s interests at every stage. Shared decision‐making ensures that patients are not only well‐informed but also actively engaged in decisions regarding their care. Equitable access to information fosters an inclusive environment, supports informed consent and autonomy, and enhances patient empowerment and the quality of care. Reports indicated improvements in data management through improved documentation and information flow. Verbal communication must be accurately supported by written documentation, particularly in critical situations such as DNAR decisions. Structured reporting methods such as checklists help make verbal exchanges more effective by reducing distractions. Changes in care plans or patient conditions must be promptly communicated to ensure the continuity of care. Efforts to inform professionals about procedural changes and use of visual aids have also been highlighted. Thorough documentation is essential to improve patient care instructions and effective information management. Data protection and confidentiality are critical in maintaining trust and ethical standards. Professionals were reminded to handle the records carefully to ensure accurate patient identification and prevent errors. The organization communicated potential data security threats to the relevant authorities. Technical measures and guidelines were implemented to securely handle devices and sensitive information, with recent facility upgrades improving patient safety and minimizing information breach risk.

“A febrile older patient with multiple comorbidities requires a routine checklist from each attending physician. This should include a medication list, hydration plan, and instructions for the following 24 h.” (Practices, Prescriptions and Recommendations)

#### 4.2.5. Enhancing Patient Care

Unified guidelines and protocols are crucial for preventing ethical incidents and ensuring consistent patient care. Insights from patient safety incident reports have highlighted the importance of understanding patient pathways and following established instructions. Regularly reviewed and standardized operational guidelines support the well‐being of patients and professionals. Effective planning prevents delays, ensures timely assistance, and avoids resource overburdens. Structured checklists, particularly during inter‐unit transfers, enhance care delivery. Detailed care plans, especially for end‐of‐life situations, help reduce unnecessary suffering and minimize confusion among professionals. To prevent ethical incidents, patient placement must be aligned with specific treatment needs, considering factors such as age and individual requirements. Incident reports have led to changes in facility layouts, improved patient safety, refined protocols, and optimized patient flow. Pain control remains a priority in medication management, reinforcing that DNAR decisions should not preclude the necessary treatments. Reports also emphasize the unique care needs of vulnerable groups. Involving family members and implementing age‐appropriate approaches are essential for pediatric and older patients. By fostering a culture of accountability through patient safety incident reporting systems, healthcare professionals are encouraged to take responsibility for delivering ethical patient care, respecting patient autonomy, and implementing improvements based on lessons learned from reported incidents.

“Adequate, appropriate, and suitable facilities for families and staff are necessary to ensure safe and proper care is provided individually while respecting the rights of the child and their families.” (Working, Infants and Children)

## 5. Discussion

The patient safety incident reporting system, enhanced by the ethical dimension, is a systematic tool for managing healthcare practices that enable the identification of deviations in ethical competence and conduct. These issues can then be addressed through discussions within healthcare settings to improve patient care quality and promote an ethical culture. We applied a data‐driven approach to analyze ethical incident reports, categorizing them with the assistance of AI and inductive content analysis, highlighting common patterns, themes, and subcategories.

We identified two overarching themes in the reported ethical incidents: (1) professional conduct, for example, issues related to communication, teamwork, and workplace behavior and (2) patient care management, for example, challenges concerning patient autonomy, decision‐making, and care quality. Actions and preventive measures to address these incidents were categorized into five themes: (1) continuous improvement; (2) enhancing professional competence; (3) fostering professional communication and conduct; (4) improving data management; and (5) enhancing patient care. Analyzing these themes in alignment with the components of the nursing paradigm, including person, environment, health, and nursing, can deepen our understanding of nursing’s role in supporting health [32]. This study deepened our understanding of managing ethical incidents in a nonblaming manner and demonstrated how healthcare organizations can support professionals in upholding their ethical values, responsibilities, and accountability.

As we proceeded with reading and analyzing research materials, an interpretive approach to the actions and prevention of ethical incidents was adopted. Specifically, the current findings do not impose rigid, universal solutions to organizing the management of ethical incident prevention, but instead present context‐sensitive strategies derived from empirical materials. In sum, themes of learning, professional development, and ethical discourse emerge as key methods in ethical incident prevention.

According to previous studies, ethical incidents are linked to organizational culture, which influences communication between professionals and interactions with patients [8]. Our findings reveal persistent issues with professional behavior that negatively affect relationships among professionals, patients, and families. Recurring inappropriate conduct, often publicly occurring between professionals from different units or disciplines, exacerbates tension. Despite efforts to improve patient safety culture and encourage incident reporting, evidence of blame among professionals persists [21]. Additionally, this study revealed that ethical incidents often arise from information flow and data management issues, particularly unclear directives, care plans, and documentation errors. This aligns with previous studies identifying poor documentation quality as a key factor in healthcare information exchange incidents [33].

Ethical incidents often involve vulnerable patient groups. Vulnerable patients often experience health issues that can be prevented but are exacerbated by inadequate care [34]. According to our results, ethical incidents are particularly common among children, adolescents, individuals with mental health conditions, critically ill or end‐of‐life patients, and older adults. Reports also emphasized the crucial role that relatives play in care. Ethical challenges faced in the care of older adults include a lack of respect, inadequate humane treatment, and difficulties in building trust [35], whereas ethical concerns in end‐of‐life care include issues with DNAR directives, treatment levels, transfers, and pain management [36]. Family members of hospitalized patients also experience vulnerability, with their perceptions of care quality shaped by healthcare professionals’ behaviors and interactions [37]. A lack of specialized expertise in caring for these groups can lead to insufficient respect, attention, or age‐appropriate care, contributing to ethical dilemmas.

Additionally, ethical incidents related to vulnerable patients also impact healthcare professionals. Pediatric nurses have expressed feelings of powerlessness when addressing ethical issues [38], while mental health nurses encounter complex situations where aligning care with ethical principles and safeguarding patient autonomy is critical [39]. Caring for dying patients induces moral distress, often due to poor communication and unmet patient wishes [36]. According to our results, ethical incidents are prevalent across the examined healthcare settings. The findings highlight the persistent nature of these issues, emphasizing the need to identify and address them. By delving deeper into the specific needs of vulnerable groups, interventions and practices can be better tailored to ensure the protection and respect of their rights while mitigating the ethical challenges healthcare professionals face. Additionally, healthcare systems should be equipped to support professionals in managing these complex ethical issues.

Consistent with a previous study [33], the recommendations for improving ethical and high‐quality patient care highlight the importance of professional conduct, workforce management, ensuring competence, and supporting professional well‐being. Efforts to promote a learning culture, establish clear guidelines, and use structured checklists to ensure consistent care support these findings [40]. However, as noted in earlier studies, a recurring challenge is the inadequate management of checklists and the implementation of guidelines. In addition, training outcomes and discussion insights are often undocumented, hindering the evaluation of their effectiveness.

As we proceeded with our analysis, organizing and developing an ethical culture in healthcare emerged as a dynamic process through open dialog and institutional support. The role of managers was central: Continuous improvement was collaboratively shaped by healthcare professionals, which fostered a participatory approach to ethical management. Our findings align with previous studies. Open dialogs about ethical incidents empower nurses to act ethically, balancing patient and societal needs while alleviating moral distress [39]. For example, pediatric nurses benefit from tailored support to navigate ethical dilemmas and enhance patient care [38]. In contrast, the care of older adults requires a comprehensive approach, standardized guidelines, and organizational structures that actively engage patients and their families [35]. By prioritizing these strategies, healthcare systems can better address moral challenges, improve outcomes for vulnerable populations, and reduce the burden on healthcare professionals. These insights form the foundation for guiding management strategies and shaping educational initiatives.

While a patient safety incident reporting system is a valuable tool for promoting an ethical organizational culture by identifying, discussing, and addressing ethical shortcomings, it has notable limitations. Consistent with previous studies, report management tends to be superficial and conducted at a general level [22]. Incident reports often fail to track whether corrective actions have been implemented or sustained, highlighting a significant gap in their effectiveness. Similarly, a previous study has found that planned improvement actions in response to incidents, although crucial, have rarely been studied in terms of their contribution to sustainable safety practices [21]. This lack of follow‐up may diminish professionals’ willingness to report incidents, limiting the system’s effectiveness, a limitation also recognized in an earlier study [40].

Ethical incidents can place a significant burden on healthcare professionals [41], potentially compromising their ability to perform their roles effectively. This burden can negatively impact nurses’ well‐being, professional performance, and job satisfaction, thereby increasing their likelihood of early career departure [42]. Supporting employee well‐being involves identifying and addressing the factors that contribute to mental and emotional strain [43]. Nursing managers, through their actions and ethical leadership, can reduce the negative impacts of ethical issues, significantly enhance job satisfaction, and foster the affective commitment of nursing staff, thereby reducing stress and burnout among healthcare professionals [18]. The insights generated by this study can assist healthcare organizations and professionals in navigating ethically challenging situations, guiding ethical decision‐making, and fostering discussions about ethics within the healthcare environment. Ultimately, these efforts can enhance the quality of care and help mitigate the moral distress and ethical burden experienced by professionals.

## 6. Strengths and Limitations

The strength of this study was its good sample size, which enhanced the validity and utility of the findings. To efficiently analyze large datasets, we adopted a new AI‐based method that provides reliable and meaningful results while saving time and reducing the workload. AI helped uncover valuable insights that might otherwise have been overlooked. Our experience with AI met our expectations and aligned with the findings of a previous study [27]. This study also had certain limitations.

The AI‐assisted analysis was conducted using Aiwo, a proprietary tool, which limits transparency of the analytic process and its underlying algorithms. Although all AI‐generated categories were critically reviewed and interpreted by the researchers, the proprietary nature of the tool remains a limitation. The first 100 most relevant free‐text entries per AI‐generated subcategory were selected based on Aiwo’s automated relevance ranking to allow a focused and feasible analysis of the most informative data. This approach provided a manageable overview of each subcategory. However, reliance on AI‐based relevance ranking may have influenced which entries were prioritized, representing a potential study limitation. Additionally, the AI analysis encountered errors, such as duplicates, misclassifications, and the inclusion of irrelevant texts. Consistent with a previous study [27], some free‐text entries were assigned to multiple subcategories, increasing the data volume. While these challenges highlight the need for human interpretation and AI support, they do not significantly affect the validity or relevance of the findings, demonstrating AI’s potential as a valuable research tool.

Including ethical incidents in the HaiPro system is a recent development, and awareness of this reporting option may not be widespread among healthcare professionals. Increased awareness in the future could influence the results if data were collected later. In addition, issues with incident reports included errors in completing the original forms, in which the text did not always align with the designated headings. Free text entries were also extracted as fragmented descriptions, often in raw terms, requiring contextual knowledge of the healthcare environment and additional analytical effort for accurate interpretation. Finally, subjective interpretations may have influenced the results because a single author conducted the analysis based on AI‐generated data.

## 7. Conclusions

The findings underscore the importance of cultivating an ethical culture in healthcare. To achieve meaningful improvement, strategic and transparent handling of ethical incidents must be prioritized and supported by structured, effective, goal‐oriented, and engaging reporting systems for staff. Concrete preventive actions should replace the retrospective addressing of incidents. Continuous improvement efforts should focus on developing seamless patient processes, prioritizing patient‐centered outcomes, particularly for vulnerable patients, and promoting inter‐unit collaboration, professional behavior, and effective team communication. These advancements rely on the active participation of healthcare professionals, fostering a participatory approach to ethical management. By providing valuable insights into a consistent and efficient structure for managing ethical incident prevention in healthcare, organizations can enhance decision‐making, ensure accountability, support professionals in resolving ethical dilemmas, and alleviate the burden associated with ethical incidents. Addressing these interconnected elements allows healthcare organizations to elevate care standards, ensure sustainable improvements in patient care quality, and create a more supportive environment for healthcare professionals.

### 7.1. Implications for Nursing Management

This study provides valuable insights for nursing managers on preventing ethical incidents in healthcare by strengthening healthcare professionals’ ethical competence and conduct and ensuring high‐quality patient care. Competent management is critical in promoting a nonblaming, reflective approach while encouraging staff to report incidents. Nursing managers are pivotal in cultivating an ethical culture within healthcare organizations. Beyond operational oversight, they guide strategic initiatives, shape educational programs, and support healthcare professionals in upholding ethical values and responsibilities. By cultivating an ethical culture and implementing preventive measures, nursing managers enhance care quality, ensure sustainability, and promote the well‐being of both patients and professionals.

## Author Contributions

Paula Järvisalo conceived and designed the study, collected and analyzed data, and drafted the initial manuscript.

Monika von Bonsdorff conceived and designed the study, supervised data collection, critically revised the manuscript, and provided feedback on the manuscript.

Kaisa Haatainen conceived and designed the study, supervised data collection, contributed to data analysis, and reviewed the manuscript.

Marja Härkänen conceived and designed the study, supervised data collection, and critically revised the manuscript for important intellectual content.

## Funding

This research received financial support for language editing from the Wellbeing Services County of North Savo (Kuopio University Hospital/Kuopion Yliopistollinen Sairaala). Open access publishing facilitated by Ita‐Suomen yliopisto, as part of the Wiley ‐ FinELib agreement.

## Conflicts of Interest

The authors declare no conflicts of interest.

## Data Availability

The research data are confidential incident reports that should not be shared with any third parties.

## References

[1] Varkey B. , Principles of Clinical Ethics and Theis Application to Practice, Medical Principles and Practice. (2021) 30, no. 1, 17–28, 10.1159/000509119. 32498071 PMC7923912

[2] Stolt M. , Leino-Kilpi H. , Ruokonen M. , Repo H. , and Suhonen R. , Ethics Interventions for Healthcare Professionals and Students: A Systematic Review, Nursing Ethics. (2017) 25, no. 2, 133–152, 10.1177/0969733017700237, 2-s2.0-85041556859. 28393607

[3] International Council of Nurses , The ICN Code of Ethics for Nurses, 2021, https://www.icn.ch/sites/default/files/2023-06/ICN_Code-of-Ethics_EN_Web.pdf.

[4] World Health Organization , Ethical Principles, 2018, https://www.who.int/about/ethics/ethical-principles.

[5] Andersson H. , Svensson A. , Frank C. , Rantala A. , Holmberg M. , and Bremer A. , Ethics Education to Support Ethical Competence Learning in Healthcare: An Integrative Systematic Review, BMC Medical Ethics. (2022) 23, no. 29, 1–26, 10.1186/s12910-022-00766-z. 35305627 PMC8933936

[6] Spronk B. , Stolper M. , and Widdershoven G. , Tragedy in Moral Case Deliberation, Med Health Care Philos. (2016) 20, no. 3, 321–333, 10.1007/s11019-016-9749-7, 2-s2.0-85001085930. PMC5569132 27913914

[7] Usberg G. , Uibu E. , Urban R. , and Kangasniemi M. , Ethical Conflicts in Nursing: An Interview Study, Nursing Ethics. (2021) 28, no. 2, 230–241, 10.1177/0969733020945751. 32909885

[8] Wehkamp K. , Kuhn E. , Petzina R. , Buyx A. , and Rogge A. , Enhancing Patient Safety by Integrating Ethical Dimensions to Critical Incident Reporting Systems, BMC Medical Ethics. (2021) 22, no. 1, 10.1186/s12910-021-00593-8. PMC7941704 33685473

[9] Choe K. , Kwon S. , and Kim S. , How do Ethically Competent Nurses Behave in Clinical Nursing Practice? A Qualitative Study, Journal of Nursing Management. (2022) 30, no. 8, 4461–4471, 10.1111/jonm.13884. 36326092

[10] Kulju K. , Suhonen R. , Puukka P. , Tolvanen A. , and Leino-Kilpi H. , Self-Evaluated Ethical Competence of a Practicing Physiotherapist: A National Study in Finland, BMC Medical Ethics. (2020) 21, no. 43, 10.1186/s12910-020-00469-3. PMC7257238 32471504

[11] Maluwa V. M. , Maluwa A. O. , Mwalabu G. , and Msiska G. , Assessment of Ethical Competence Among Clinical Nurses in Health Facilities, Nursing Ethics. (2021) 29, no. 1, 181–193, 10.1177/09697330211010259. 34346258

[12] Asgari S. , Shafipour V. , Taraghi Z. , and Yazdani-Cherati J. , Relationship Between Moral Distress and Ethical Climate With Job Satisfaction in Nurses, Nursing Ethics. (2017) 26, no. 2, 346–356, 10.1177/0969733017712083, 2-s2.0-85041283828. 28718349

[13] Kulju K. , Stolt M. , Suhonen R. , and Leino-Kilpi H. , Ethical Competence, Nursing Ethics. (2015) 23, no. 4, 401–412, 10.1177/0969733014567025, 2-s2.0-84973497810. 25670176

[14] Ghezeljeh T. N. , Farahani M. A. , and Ladani F. K. , Factors Affecting Nursing Error Communication in Intensive Care Units: A Qualitative Study, Nursing Ethics. (2020) 28, no. 1, 131–144, 10.1177/0969733020952100. 32985367

[15] Dalmolin G. L. , Lanes T. C. , Bernardi C. M. S. , and Ramos F. R. S. , Conceptual Framework for the Ethical Climate in Health Professionals, Nursing Ethics. (2022) 29, no. 5, 1174–1185, 10.1177/09697330221075741. 35545250

[16] Simha A. and Pandey J. , Trust, Ethical Climate and Nurses’ Turnover Intention, Nursing Ethics. (2020) 28, no. 5, 714–722, 10.1177/0969733020964855. 33190580

[17] Gilvari T. , Abbaszadeh A. , Borhani F. et al., Relationship of the Hospital Ethical Climate With Nurses’ Attitude to Interprofessional Collaboration, Journal of Clinical and Diagnostic Research. (2019) 13, no. 11, 16–19.

[18] Gonzalez-Garcıa A. , Pinto-Carral A. , Marques-Sanchez P. et al., Characteristics of the Competency Ethical Principles for the Nurse Manager: A Systematic Review, Journal of Advanced Nursing. (2025) 81, 1717–1733. 40223884 10.1155/jonm/2575609 PMC11985233

[19] Manninen K. , Björling G. , Kuznecova J. , and Lakanmaa R. L. , Ethical Coffee Room: An International Collaboration in Learning Ethics Digitally, Nursing Ethics. (2020) 27, no. 8, 1655–1668, 10.1177/0969733020934145. 32666868

[20] Serou N. , Sahota L. M. , Husband A. K. , Forrest S. P. , Slight R. D. , and Slight S. P. , Learning From Safety Incidents in High-Reliability Organizations: A Systematic Review of Learning Tools That Could Be Adapted and Used in Healthcare, International Journal for Quality in Health Care. (2021) 33, no. 1, 10.1093/intqhc/mzab046. PMC8271183 33729493

[21] Saarikoski T. , Haatainen K. , Roine R. , and Turunen H. , Significant Differences in the Quality of Incident Reports—A Comparison of Four Acute Hospitals in Finland, Saf Reliab. (2023) 41, no. 4, 225–237, 10.1080/09617353.2022.2154023.

[22] Hiltunen T. , Suhonen R. , Inkilä J. , and Leino-Kilpi H. , Reporting and Managing Ethical Issues in Intensive Care Using the Critical Incident Reporting System, Nursing Ethics. (June 2024) 32. 10.1177/09697330241244514 PMC11771081 38847389

[23] Awanic. Haipro, 2024, https://awanic.fi/haipro/.

[24] Liukka M. , Hupli M. , and Turunen H. , How Transformational Leadership Appears in Action With Adverse Events? A Study for Finnish Nurse Manager, Journal of Nursing Management. (September 2018) 26, no. 6, 639–646, 10.1111/jonm.12592, 2-s2.0-85038898587. 29277948

[25] Specchia M. L. , Cozzolino M. R. , Carini E. et al., Leadership Styles and Nurses’ Job Satisfaction. Results of a Systematic Review, IJERPH. (February 2021) 18, no. 4, 10.3390/ijerph18041552. PMC7915070 33562016

[26] Jalali M. S. and Akhavan A. , Integrating AI Language Models in Qualitative Research: Replicating Interview Data Analysis With ChatGPT, System Dynamics Review. (2024) 40, no. 3, 10.1002/sdr.1772.

[27] Härkänen M. , Haatainen K. , Vehviläinen-Julkunen K. , and Miettinen M. , Artificial Intelligence for Identifying the Prevention of Medication Incidents Causing Serious or Moderate Harm: An Analysis Using Incident Reporters’ Views, International Journal of Environmental Research and Public Health. (2021) 18, no. 17. 10.3390/ijerph18179206 PMC8431329 34501795

[28] Devlin J. , Chang M. W. , Lee K. et al., BERT: Pre-Training of Deep Bidirectional Transformers for Language Understanding, NAACL-HLT. (2019) 1, 4171–4186.

[29] Aiwo.ai , Why Should Organizations Change From Text Analytics to Aiwo’s Qualitative Analytics?, 2020, https://aiwo.ai/miksi-organisaatioiden-tulisi-vaihtaa-tekstianalytiikka-aiwon-laadulliseen-analytiikkaan/.

[30] Lindgren B. M. , Lundman B. , and Graneheim U. H. , Abstraction and Interpretation During the Qualitative Content Analysis Process, International Journal of Nursing Studies. (August 2020) 108, 10.1016/j.ijnurstu.2020.103632. 32505813

[31] Finnish National Board on Research Integrity (TENK) , The Ethical Principles of Research With Human Participants and Ethical Review in the Human Sciences in Finland, 2019, https://tenk.fi/sites/default/files/2021-01/Ethical_review_in_human_sciences_2020.pdf.

[32] Nikfarid L. , Hekmat N. , Vedad A. , and Rajabi A. , The Main Nursing Metaparadigm Concepts in Human Caring Theory and Persian Mysticism: A Comparative Study, Journal of Medical Ethics and History of Medicine. (2018) 11, no. 6. PMC6150916 30258556

[33] Hyvämäki P. , Sneck S. , Meriläinen M. et al., Interorganizational Health Information Exchange-Related Patient Safety Incidents: A Descriptive Register-Based Qualitative Study, International Journal of Medical Informatics. (2023) 174. 10.1016/j.ijmedinf.2023.105045 36958225

[34] Waisel D. B. , Vulnerable Populations in Healthcare, Current Opinion in Anaesthesiology. (2013) 26, no. 2, 186–192, 10.1097/aco.0b013e32835e8c17, 2-s2.0-84874949051. 23385323

[35] Zendehtalab H. , Vanaki Z. , and Memarian R. , Ethical Challenges in Caring for Healthy Older Adults: Qualitative Perspectives, Nursing Ethics. (2023) 30, no. 4, 542–555, 10.1177/09697330221081953. 36841931

[36] De B. E. L. , Giannetta N. , Ercolani S. et al., Nurses’ Moral Distress in End-of-Life Care: A Qualitative Study, Nursing Ethics. (2020) 28, no. 5, 614–627, 10.1177/0969733020964859. 33267730

[37] Nygaard A. M. , Haugdahl H. S. , Laholt H. , Brinchmann B. S. , and Lind R. , Professionals’ Narratives of Interactions With Patients’ Families in Intensive Care, Nursing Ethics. (2022) 29, no. 4, 885–898, 10.1177/09697330211050995. 35196935 PMC9289990

[38] Schulz I. , O’Neill J. , Gillam P. , and Gillam L. , The Scope of Ethical Dilemmas in Paediatric Nursing: A Survey of Nurses From a Tertiary Paediatric Centre in Australia, Nursing Ethics. (2023) 30, no. 4, 526–541, 10.1177/09697330231153916. 36877536

[39] Ventura C. A. A. , Austin W. , Carrara B. S. , and de Brito E. S. , Nursing Care in Mental Health: Human Rights and Ethical Issues, Nursing Ethics. (2021) 28, no. 4, 463–480, 10.1177/0969733020952102. 33111635

[40] Liukka M. , Hupli M. , and Turunen H. , Problems With Incident Reporting: Reports Lead Rarely to Recommendations, Journal of Clinical Nursing. (May 2019) 28, no. 9–10, 1607–1613, 10.1111/jocn.14765, 2-s2.0-85060238875. 30589957

[41] Hopia H. and Heino-Tolonen T. , Families in Paediatric Oncology Nursing: Critical Incidents From the Nurses’ Perspective, Journal of Pediatric Nursing. (2019) 44, 28–35, 10.1016/j.pedn.2018.10.013, 2-s2.0-85055283605. 30528181

[42] Feng H. , Zhang M. , Li X. , and Shen Y. , The Level and Outcomes of Emotional Labor in Nurses: A Scoping Review, Journal of Nursing Management. (2024) 2024, no. 1, 10.1155/2024/5317359. PMC11919068 40224865

[43] Kakemam E. , Kalhor R. , Khakdel Z. et al., Occupational Stress and Cognitive Failure of Nurses and Associations With Self‐Reported Adverse Events: A National Cross‐Sectional Survey, Journal of Advanced Nursing. (2019) 75, no. 12, 3609–3618, 10.1111/jan.14201. 31531990

